# 

**Published:** 1919-04

**Authors:** 


### Old Jokes That We Have Met.

But How Was He Cured?—"I'm troubled with a buzzing noise in my ears all the time."

"Have you any idea as to the cause?"

"Yes, my wife wants an auto."

---

Its Failure—Doctor—Madame, did my medicine have a soporific effect?

Patient—No, doctor; it only put me to sleep.

---

Getting It All.—The doctor told him he needed carbo-hydrates, proteins and, above all something nitrogeneous. The doctor mentioned a long list of foods for him to eat. He staggered out and wabbled into a Penn avenue restaurant.

"How about breakfast?" he asked the waiter. "Is that nitrogeneous?"

The waiter didn't know.

"Are fried potatoes rich in carbo-hydrates or not?"

The waiter couldn't say.

"Well, I'll fix it," declared the poor man in despair. "Bring me a large plate of hash."—Pittsburgh Post.

---

Answered.—"Doctor, why is it that some people who are perfect wrecks live longer than others who are strong and well?"

"Er—well—you see, the others die first."—Boston Transcript.

---

His Treat—Bacon—Been to see the doctor?

Egbert—Sure thing.

"Did he treat you?"

"Oh, no; it was my treat. It cost me two dollars."

---

Why He Returned.—"Back home again, eh, doctor? What was the trouble, too healthy for you out there?"

"Exactly. There was only one case of sickness in town the whole time I was there?"

"And I suppose some other doctor had that?"

"No, I had it. It was homesickness."

